# Supplementary material for: Universality in volume-law entanglement of scrambled pure quantum states
Source: Nat Commun. 2018 Apr 24;9:1635. doi: 10.1038/s41467-018-03883-9 (PMC5915398; doi:10.1038/s41467-018-03883-9)
Supplement: Supplementary file 1 — Supplementary Information [file 41467_2018_3883_MOESM1_ESM.pdf]

Supplementary Information of Universality in volume-law entanglement of scrambled pure quantum states

SUPPLEMENTARY FIGURES

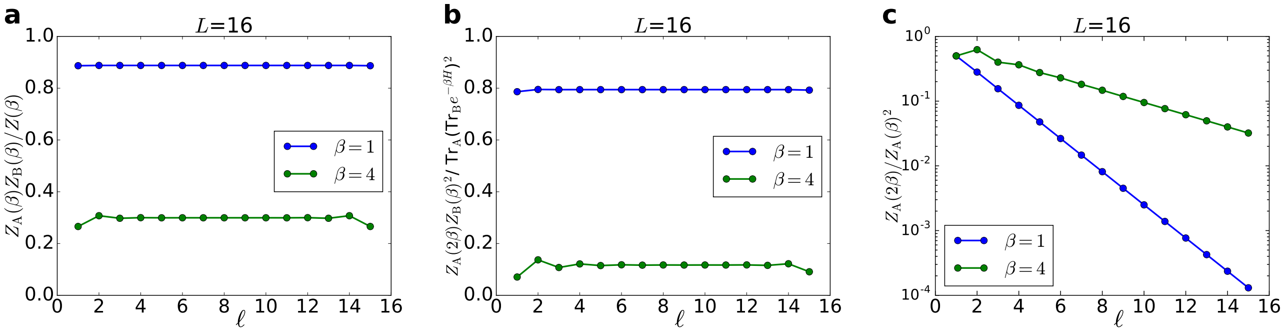

Supplementary Figure 1. **Numerical illustrations of the validity of the approximations in the derivation of the volume-law formula**. Just for completeness, we numerically examine the approximations in the derivation of the formula (5) such as $Z(\beta) \propto Z_{\mathrm{A}}(\beta)Z_{\mathrm{B}}(\beta)$, $\mathrm{tr}_{\mathrm{A}}\left(\mathrm{tr}_{\mathrm{B}}\, e^{-\beta H}\right)^2 \propto Z_{\mathrm{A}}(2\beta)Z_{\mathrm{B}}^2(\beta)$, and $Z_{\mathrm{A}}(2\beta)/Z_{\mathrm{A}}^2(\beta) \propto \exp(-\ell)$, where the proportional constants depend only on β and not on ℓ. These assumptions are basically valid for $1 \ll \ell \ll L$. Numerical data of (**a**) $Z_{\mathrm{A}}(\beta)Z_{\mathrm{B}}(\beta)/Z(\beta)$, (**b**) $Z_{\mathrm{A}}(2\beta)Z_{\mathrm{B}}^2(\beta)/\,\mathrm{tr}_{\mathrm{A}}\left(\mathrm{tr}_{\mathrm{B}}\, e^{-\beta H}\right)^2$, and (**c**) $Z_{\mathrm{A}}(2\beta)/Z_{\mathrm{A}}^2(\beta)$ for the XX chain ((Eq. (9) in the main text)) of $L = 16$ at inverse temperature $\beta = 1, 4$ are presented. As clearly seen from the figures, $Z_{\mathrm{A}}(\beta)Z_{\mathrm{B}}(\beta)/Z(\beta)$ and $Z_{\mathrm{A}}(2\beta)Z_{\mathrm{B}}^2(\beta)/\,\mathrm{tr}_{\mathrm{A}}\left(\mathrm{tr}_{\mathrm{B}}\, e^{-\beta H}\right)^2$ do not depend on ℓ for $2 \lesssim \ell \lesssim L-2$. Also $Z_{\mathrm{A}}(2\beta)/Z_{\mathrm{A}}^2(\beta) \propto \exp(-\ell)$ holds well down to $\ell = 3$.

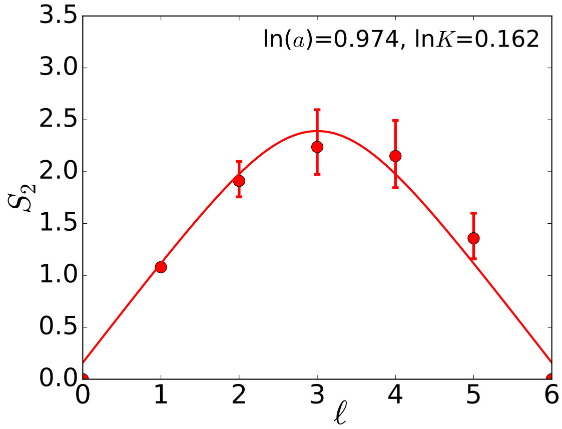

Supplementary Figure 2. **Fitting of the experiential data of the 2RPC (Fig. 4A of Ref. [1])**. As discussed in the main text, Kaufman *et al.* experimentally measured the 2RPC after quantum quench in Ref. [1]. We perform a fitting of the experimental data by our formula (5) and it yields $\ln(a) = 0.974, \ln K = 0.162$. The data are extracted from the Fig. 4A of Ref. [1] by us and all data points ($\ell = 1, \ldots, 6$) are used in the fitting. We do not take into account the error bar of each point in the fitting. The difference between the experimental data and our fitting is relatively large at $\ell = 5$. Theoretically speaking, the Page curve in a translationally invariant system is always symmetric under $\ell \leftrightarrow L - \ell$. However, the measurement of a purity in a large subsystem size is experimentally much harder than that in a small one, which probably results in the difference.

SUPPLEMENTARY NOTES

Supplementary Note 1: Functional form of mutual information

Kaufman *et al.* also measured the second Rényi mutual information (MI) between two subsystems as well as the second Rényi entropy of a single subsystem [1]. In this note we present a functional form for MI and show that the qualitative behavior of that function is the same as the one observed in the experiment.

The second Rényi MI between two subsystems A and B is defined as

$$I_2(\mathrm{A},\mathrm{B}) := S_2^{\mathrm{A}} + S_2^{\mathrm{B}} - S_2^{\mathrm{A}\cup\mathrm{B}}, \tag{1}$$

where S_2^X is the second Rényi entropy of a subsystem X. MI is known to properly count the correlations between two subsystems for excited states. Let us choose subsystems A and B which consist of $\ell_a \times M$ sites and $\ell_b \times M$ sites, respectively. We consider both cases where A and B share a boundary (i.e. $\mathrm{A}\cup\mathrm{B}$ is connected) and where they do not ($\mathrm{A}\cup\mathrm{B}$ is disconnected). According to our result in the main text, the 2REEs of these subsystems are written as

$$S_2^{\mathrm{A}} = -\ln(a^{-\ell_a} + a^{-L+\ell_a}) + \ln K, \tag{2}$$

$$S_2^{\mathrm{B}} = -\ln(a^{-\ell_b} + a^{-L+\ell_b}) + \ln K, \tag{3}$$

$$S_2^{\mathrm{A}\cup\mathrm{B}} = -\ln(a^{-(\ell_a+\ell_b)} + a^{-L+(\ell_a+\ell_b)}) + (2-q)\ln K, \tag{4}$$

where $q = 1$ when $\mathrm{A}\cup\mathrm{B}$ is connected and $q = 0$ when $\mathrm{A}\cup\mathrm{B}$ is disconnected (we note that $\ln K$ comes from the boundary of the subsystem(s)). Thus, the MI between A and B reads

$$I_2 = \ln\left(\frac{a^{-(\ell_a+\ell_b)} + a^{-L+(\ell_a+\ell_b)}}{(a^{-\ell_a} + a^{-L+\ell_a})(a^{-\ell_b} + a^{-L+\ell_b})}\right) + q\ln K. \tag{5}$$

For simplicity, let us take $\ell_a = \ell_b = \ell/2$ where ℓ denotes a combined volume of the subsystems. Then the above formula reduces to

$$I_2 = \ln\left(\frac{a^{-\ell} + a^{-L+\ell}}{(a^{-\ell/2} + a^{-L+\ell/2})^2}\right) + q\ln K. \tag{6}$$

For $(1 \ll)\,\ell \ll L/2$, I_2 can be approximated as $I_2 \approx a^{-L+\ell}\cdot(a^{\ell}-2) + q\ln K$, which means that I_2 grows exponentially with ℓ. For $L/2 \ll \ell\,(\ll L)$, one can find $I_2 \approx (2\ell - L)\ln a - 2\ln(1 + a^{\ell-L}) + \ln(1 + a^{L-2\ell}) + q\ln K$, which means a linear growth of I_2 with ℓ. Those behaviors are quite similar to the result of the experiment (Fig. 4C of Ref. [1]), although the experimental data were collected as the mean of all configurations of the subsystems whose combined volume is ℓ.

Supplementary Note 2: Applicability of our formula for general scrambled states

Here we present possible explanations on the applicability of our formula (5) to general eigenstates of non-integrable Hamiltonians and the time-averaged 2RPC after quantum quenches.

For a given pure state $|\psi\rangle = \sum_i c_i |i\rangle$, where $\{|i\rangle\}_i$ is a complete basis of the system, one obtains

$$S_2 = -\ln\left(\mathrm{tr}_{\mathrm{A}}\,\rho_{\mathrm{A}}^2\right) = -\ln\left(\mathrm{tr}_{\mathrm{A}}\left(\sum_{i,j,k,l} c_i c_j^* c_k c_l^*\,\mathrm{tr}_{\mathrm{B}}\left(|i\rangle\langle j|\right)\mathrm{tr}_{\mathrm{B}}\left(|k\rangle\langle l|\right)\right)\right). \tag{7}$$

We can decompose the summation into that of diagonal ensembles and others as

$$S_2 = -\ln\left[\mathrm{tr}_{\mathrm{A}}\left(\mathrm{tr}_{\mathrm{B}}\left(\hat{\rho}_{\mathrm{dia}}\right)^2\right) + \mathrm{tr}_{\mathrm{B}}\left(\mathrm{tr}_{\mathrm{A}}\left(\hat{\rho}_{\mathrm{dia}}\right)^2\right) + I_{\mathrm{off}}\right], \tag{8}$$

where the diagonal ensemble in the present basis is defined as

$$\hat{\rho}_{\mathrm{dia}} \equiv \sum_i |c_i|^2 |i\rangle\langle i|, \tag{9}$$

$$I_{\mathrm{off}} \equiv \mathrm{tr}_{\mathrm{A}}\left(\sum_{(i,j,k,l)}{}' c_i c_j^* c_k c_l^*\,\mathrm{tr}_{\mathrm{B}}\left(|i\rangle\langle j|\right)\mathrm{tr}_{\mathrm{B}}\left(|k\rangle\langle l|\right)\right) \tag{10}$$

(the summation $\sum'$ runs all (i,j,k,l) other than those satisfying $(i,k)=(j,l)$ or $(i,k)=(l,j)$). When $I_{\rm off}$ can be neglected, and $\rho_{\rm diag}$ has extensiveness, i.e. $\log(\mathrm{tr_A}\left(\mathrm{tr_B}\left(\hat{\rho}_{\rm dia}\right)^2\right))\propto -\ell$, the above expression reduces to the same form as Eq. (5) in the main text. Namely, the 2RPC of $|\psi\rangle$ obeys the formula (5), when these conditions are satisfied. For the cTPQ states, the off-diagonal contribution is a sum of the products of uncorrelated zero-mean random numbers. It naturally dies out for sufficiently large systems even without taking the random number average due to the central limit theorem. The observed applicability of our formula (5) to the energy eigenstates of non-integrable models indicates that, in non-integrable models, the coefficients $\{c_i\}_i$ can be regarded as a sort of random numbers just as cTPQ states. Then, the off-diagonal term $I_{\rm off}$ in Supplementary Eq. (8) vanishes and we obtain

$$\overline{S_2}=-\ln\left[\mathrm{tr_A}(\mathrm{tr_B}\,\rho_{\rm diag})^2+\mathrm{tr_B}(\mathrm{tr_A}\,\rho_{\rm diag})^2\right]. \tag{11}$$

Therefore, (5) holds when $\ln[\mathrm{tr_A}(\mathrm{tr_B}(\rho_{\rm diag})^2)]$ is extensive. Conversely, the breakdown of our formula (5) for integrable models means that an infinitely large number of conserved quantities in such models strongly constraint the property of the coefficients, and we can no longer regard them as zero-mean random numbers. Although the above argument seems to depend on the choice of the basis, the 2RPC and thereby the applicability of our formula (5) should not be sensitive to that. Therefore, we expect that the randomness of the coefficients is a common feature among any, presumably local, basis. Regarding the point of the extensiveness of $\ln[\mathrm{tr_A}(\mathrm{tr_B}(\rho_{\rm diag})^2)]$ of the energy eigenstates in the non-integrable model, $\ln[\mathrm{tr_A}(\mathrm{tr_B}(\rho_{\rm diag})^2)]$ may be different from a linear function. In this case, we can employ Supplementary Eq. (11) as a fitting function. Here, $\rho_{\rm diag}$ has to be chosen appropriately, for example, the diagonal ensemble proposed in Ref. [2]. Substituting another $\rho_{\rm diag}$ to Supplementary Eq. (11), we obtain another formula to describe the Page curve of this class of pure quantum states.

For the time-average of 2RPC, we can deepen the discussion by taking the eigenstates of the system as a basis $\{|i\rangle\}$ and explicitly calculating the time-average. The time-average of the 2REE $S_2(t)$ is defined as $\overline{S_2}^{\rm time}:=\lim_{T\to\infty}\frac{1}{T}\int_0^T dtS_2(t)=-\lim_{T\to\infty}\frac{1}{T}\int_0^T dt\ln(\mathrm{tr_A}(\rho_{\rm A}(t))^2)$, but here we consider a slightly different time-average $\overline{S_2}=-\ln\left(\lim_{T\to\infty}\frac{1}{T}\int_0^T dt\,\mathrm{tr_A}(\rho_{\rm A}(t))^2\right)$. If one assumes that $S_2(t)$ reaches to a stationary value at $t\to\infty$, these two time-averages coincide with each other. We expand the initial state of the quench $|\psi(0)\rangle$ in terms of the eigenstates of the Hamiltonian after the quench as $|\psi(0)\rangle=\sum_n c_n|n\rangle$. Then, it is easy to trace its time evolution after the quench, $|\psi(t)\rangle=\sum_n c_n e^{-iE_nt}|n\rangle$, and write down the density matrix for $t>0$, $\rho(t)=|\psi(t)\rangle\langle\psi(t)|=\sum_{n,m}c_nc_m^*e^{-i(E_n-E_m)t}|n\rangle\langle m|$. By taking the partial trace of the subsystem B, we obtain the reduced density matrix, $\rho_{\rm A}(t)=\mathrm{tr_B}\,\rho(t)=\sum_{n,m}c_nc_m^*e^{-i(E_n-E_m)t}\rho_{\rm A}(n;m)$ where $\rho_{\rm A}(n;m):=\mathrm{tr_B}\left(|n\rangle\langle m|\right)$, and the time-averaged entropy, $\overline{S_2}=-\ln\left(\sum'_{n,m,k,l}c_nc_m^*c_kc_l^*\;\mathrm{tr_A}\left(\rho_{\rm A}(n;m)\rho_{\rm A}(k;l)\right)\right)$. Here the summation is taken over the indices satisfying $E_n-E_m+E_k-E_l=0$. In a finite size system with discrete energy spectrum, there is almost no chance to satisfy this condition except for the trivial solutions $(n=m;\,k=l)$ or $(n=l;\,k=m)$ [3]. If we assume that the dominant contribution to $\overline{S_2}$ comes from these trivial ones and recall the definition of the diagonal ensemble for the energy eigenstates, $\rho_{\rm diag}=\sum_n|c_n|^2|n\rangle\langle n|$, we reach a simple expression:

$$\overline{S_2}=-\ln\left[\mathrm{tr_A}(\mathrm{tr_B}\,\rho_{\rm diag})^2+\mathrm{tr_B}(\mathrm{tr_A}\,\rho_{\rm diag})^2\right]. \tag{12}$$

Compared with Supplementary Eq. (8), the time-averaged S_2 after the quench does not contain the off-diagonal term $I_{\rm off}$.

Supplementary Note 3: Extension of Eq. (5) to disordered systems

Here we present a discussion on the extension of our result to disordered systems which have quenched randomness. Our derivation in the manuscript still holds up to Eq. (4) in disordered systems. In between Eq. (4) and (5), we assume that $-\ln Z_{\rm A,B}$ is proportional to the volume of the corresponding subregion. This assumption does not hold in disordered systems, so we need a more careful calculation in this point. Since the disorders are quenched at each site, we assume that the partition functions are written as follows:

$$Z_{\rm A}(\beta)=q_1\prod_{i\in{\rm A}}\hat{x}_i,\; Z_{\rm B}(\beta)=q_1\prod_{i\in{\rm B}}\hat{x}_i, \tag{13}$$

$$Z_{\rm A}(2\beta)=q_2\prod_{i\in{\rm A}}\hat{y}_i,\; Z_{\rm B}(2\beta)=q_2\prod_{i\in{\rm B}}\hat{y}_i, \tag{14}$$

where $\hat{x}_i$ and $\hat{y}_i$ are i.i.d random variables whose randomness stems from the disorders and q_1 and q_2 are some constants. One can check that this assumption holds in, e.g., the Ising chain with random magnetic field. When we

write

$$\hat{a}_i \equiv \frac{\hat{x}_i^2}{\hat{y}_i}, \;\; Q(\beta) = \frac{q_1^2}{q_2}, \tag{15}$$

Eq. (5) for disordered systems becomes

$$S_2(\ell) = \sum_{i\in \mathrm{A}} \ln \hat{a}_i - \ln\left(1 + \prod_{i\in\mathrm{A}} \hat{a}_i \prod_{i\in\mathrm{B}} \frac{1}{\hat{a}_i}\right) + \ln K(\beta), \tag{16}$$

where $K \equiv R/Q$. Then, the random average of S_2 over disorder realizations gives the same functional form as Eq. (5) (since $\hat{a}_i$ is i.i.d, $\prod_{i\in\mathrm{A}} \hat{a}_i \prod_{i\in\mathrm{B}} \frac{1}{\hat{a}_i}$ converges to $a^{2\ell-L}$, where a is the average of $\{\hat{a}_i\}$ without the random average).

Supplementary Note 4: Proof on the difference between $\overline{S}_n$ and $\tilde{S}_n$

In this note we provide a proof of the following property mentioned in the Method section:

$$\overline{\log\left[\mathrm{tr}_{\mathrm{A}}\left(\rho_{\mathrm{A}}^n\right)\right]} = \log \overline{\left[\mathrm{tr}_{\mathrm{A}}\left(\rho_{\mathrm{A}}^n\right)\right]} + O(1/d), \tag{17}$$

where $d = \alpha^L$, L is the system size, and $1 < \alpha$ is the effective dimension of the system. $\alpha = 2$ at infinite temperature for $S = 1/2$ spin systems. We note that another proof is provided in Ref. [4]

Sketch of the proof

Let us set up the notations. We denote $W[z,\bar{z}] \equiv \mathrm{tr}_{\mathrm{A}}\left(\rho_{\mathrm{A}}^n\right)$, where z is the random complex number whose real and imaginary parts are taken from the normal distribution. We also write the random number average of $W[z,\bar{z}]$ over the random number z as $\Omega \equiv \overline{W[z,\bar{z}]}$. Our goal is to prove

$$\overline{\log W[z,\bar{z}]} = \log \Omega + O(1/d) \iff \overline{\log\left[\frac{W[z,\bar{z}]}{\Omega}\right]} = O(1/d). \tag{18}$$

Now we formally expand the logarithm around $\frac{W[z,\bar{z}]}{\Omega} = 1$ and reach

$$\overline{\log\left[\frac{W[z,\bar{z}]}{\Omega}\right]} = -\frac{1}{2}\overline{\left(\frac{W[z,\bar{z}]}{\Omega} - 1\right)^2} + \frac{1}{3}\overline{\left(\frac{W[z,\bar{z}]}{\Omega} - 1\right)^3} - \frac{1}{4}\overline{\left(\frac{W[z,\bar{z}]}{\Omega} - 1\right)^4} + \cdots. \tag{19}$$

Here we used $\overline{\frac{W[z,\bar{z}]}{\Omega}} = 1$. The first term $\overline{\left(\frac{W[z,\bar{z}]}{\Omega} - 1\right)^2}$ gives

$$\overline{\left(\frac{W[z,\bar{z}]}{\Omega} - 1\right)^2} = \frac{\overline{W^2} - \Omega^2}{\Omega^2}, \tag{20}$$

and $\overline{W^2} - \Omega^2$ gives $\Omega^2 \times O(1/d)$. Likewise, we can see that the terms like $\overline{(W-\Omega)^m}$ only scales as $\Omega^m \times O(1/d^{\lfloor m/2 \rfloor})$ (we revisit this point later):

$$\frac{\overline{(W-\Omega)^m}}{\Omega^m} = O(1/d^{\lfloor m/2 \rfloor}). \tag{21}$$

Therefore, by summing up all the contributions, we will have

$$\overline{\log\left[\frac{W[z,\bar{z}]}{\Omega}\right]} = \sum_{l=1}^{\infty} a_l d^{-l}, \tag{22}$$

where a_l is independent of d and scales exponentially in l as seen from the direct computation. Then, for sufficiently large $d\;(= O(e^L))$, the right hand side of the above equation converges, which is order $O(1/d)$.

Rigorous Proof

The above argument is mathematically not rigorous. Since the random numbers are taken from the normal distribution, there is always a chance that $\frac{W[z,\bar{z}]}{\Omega}$ becomes larger than two, the convergence radius of the logarithm. To make the above argument rigorous, here we introduce the probability distribution for $\frac{W[z,\bar{z}]}{\Omega}$ and use the finite order Taylor expansion.

Let us write the probability distribution of $\Phi = W[z,\bar{z}]/\Omega$ to be $P[\Phi]$, so that we have

$$\overline{\log\left[\frac{W[z,\bar{z}]}{\Omega}\right]} = \int_{1/d_{\rm A}^{n-1}}^{d_{\rm A}^{n-1}} d\Phi P[\Phi] \log \Phi. \tag{23}$$

We take the integration range from $1/d_{\rm A}^{n-1}$ to $d_{\rm A}^{n-1}$ since by construction $1/d_{\rm A}^{n-1} \leqslant W[z,\bar{z}] = {\rm tr}_{\rm A}\left(\rho_{\rm A}^n\right) \leqslant 1$ and $1/d_{\rm A}^{n-1} \leqslant \Phi \leqslant d_{\rm A}^{n-1}$, where $d_{\rm A}$ is the dimension of the subsystem A. Here we assume the subsystem A is smaller than the rest of the system, ${\rm B} = \overline{\rm A}$ (when A is larger than B then the bound is given by $d_{\rm B}$). Now we expand $\log\Phi = (\Phi-1) - (\Phi-1)^2/(2\xi^2)$, where ξ is in between 1 and Φ (the Taylor theorem),

$$\overline{\log\Phi} = \int_{1/d_{\rm A}^{n-1}}^{d_{\rm A}^{n-1}} d\Phi P[\Phi](\Phi-1) - \frac{1}{2}\int_{1/d_{\rm A}^{n-1}}^{d_{\rm A}^{n-1}} d\Phi P[\Phi]\frac{(\Phi-1)^2}{\xi^2}, \tag{24}$$

but the first term gives zero because $\overline{\Phi-1} = 0$. In the following we divide the range of integration into two parts, $[1/d_{\rm A}^{n-1}, 1/2]$ and $[1/2, d_{\rm A}^{n-1}]$, and evaluate each of them.

a. Integration range $[1/d_{\rm A}^{n-1}, 1/2]$ First we would like to evaluate

$$I_1 \equiv \int_{1/d_{\rm A}^{n-1}}^{1/2} d\Phi P[\Phi]\frac{(\Phi-1)^2}{\xi^2} \geq 0. \tag{25}$$

Because $\xi > 1/d_{\rm A}^{n-1}$, we have

$$I_1 < \int_{1/d_{\rm A}^{n-1}}^{1/2} d\Phi P[\Phi] d_{\rm A}^{2(n-1)}(\Phi-1)^2, \tag{26}$$

and also because $(\Phi-1)^2 < 1$,

$$I_1 < \int_{1/d_{\rm A}^{n-1}}^{1/2} d\Phi P[\Phi] d_{\rm A}^{2(n-1)}(\Phi-1)^2 < \int_{1/d_{\rm A}^{n-1}}^{1/2} d\Phi P[\Phi] d_{\rm A}^{2(n-1)}. \tag{27}$$

This quantity has an upper bound from the Chebyshev inequality for higher moments. The inequality on the $2n$-th moment tells that ${\rm Prob}\left(|\Phi-1| > 1/2\right) \leq 2^{2n}\overline{(\Phi-1)^{2n}}$ so we obtain

$$I_1 < 2^{2n} d_{\rm A}^{2(n-1)} \times \overline{(\Phi-1)^{2n}} = O(1/d), \tag{28}$$

where we have used Supplementary Eq. (21) and $d_{\rm A} \leqslant d^{1/2}$.

b. Integration range $[1/2, d_{\rm A}^{n-1}]$ What to evaluate is

$$I_2 \equiv \int_{1/2}^{d_{\rm A}^{n-1}} d\Phi P[\Phi]\frac{(\Phi-1)^2}{\xi^2} \geq 0. \tag{29}$$

Because $\xi > 1/2$ we have

$$I_2 < 4 \times \int_{1/2}^{d_{\rm A}^{n-1}} d\Phi P[\Phi](\Phi-1)^2. \tag{30}$$

Also,

$$I_2 < 4 \times \int_{1/2}^{d_{\rm A}^{n-1}} d\Phi P[\Phi](\Phi-1)^2 < 4\overline{(\Phi-1)^2} = O(1/d), \tag{31}$$

where the last equality again comes from Supplementary Eq. (21).

Finally, by summing up the above two results, we reach

$$\overline{\log\Phi} = O(1/d), \tag{32}$$

which is the desired result.

Comments on Supplementary Eq. (21)

We have not given any proof of Supplementary Eq. (21), since proving this in full generality is too complicated. The proof goes the same as in deriving the result of the average of the Rényi entropy (just contracting the indices in the random number z), and when $m = 2$ and $n = 2$ for example we have

$$\overline{\left(W[z, \bar{z}] - \Omega\right)^2} = \sum_{ijklmop} Z^{ij}_{op} Z^{ij}_{kl} Z^{mn}_{kl} Z^{mn}_{op} + \mathrm{tr}_{\mathrm{B}} \left[\mathrm{tr}_{\mathrm{A}}\, Z^2 \left(\mathrm{tr}_{\mathrm{A}}\, Z\right)^2 \right] + (\mathrm{A} \leftrightarrow \mathrm{B}) \tag{33}$$

where $Z \equiv e^{-\beta H}$, taking indices in the subspace H_{A} (upper) and H_{B} (lower), respectively. By following the argument in the main text to pull out the extensive contributions, one can see the terms in the right hand side divided by Ω^2 are of the order of $O(1/d)$.

SUPPLEMENTARY REFERENCES

[1] Adam M. Kaufman, M. Eric Tai, Alexander Lukin, Matthew Rispoli, Robert Schittko, Philipp M. Preiss, and Markus Greiner, "Quantum thermalization through entanglement in an isolated many-body system," Science **353**, 794–800 (2016).

[2] Anatoly Dymarsky, Nima Lashkari, and Hong Liu, "Subsystem eigenstate thermalization hypothesis," Phys. Rev. E **97**, 012140 (2018).

[3] Peter Reimann, "Foundation of statistical mechanics under experimentally realistic conditions," Phys. Rev. Lett. **101**, 190403 (2008).

[4] Tsung-Cheng Lu and Tarun Grover, "Renyi entropy of chaotic eigenstates," Preprint at http://arxiv.org/abs/1709.08784 (2017).
