# Supplementary material for: Universality in volume-law entanglement of scrambled pure quantum states
Source: Nat Commun. 2018 Apr 24;9:1635. doi: 10.1038/s41467-018-03883-9 (PMC5915398; doi:10.1038/s41467-018-03883-9)
Supplement: Supplementary file 2 — Description of Additional Supplementary Files [file 41467_2018_3883_MOESM2_ESM.pdf]

Description of Additional Supplementary Files

File Name: Supplementary Movie 1

Description: **Dynamics of the second Rényi Page curve in a non-integrable system**. Dynamics of the 2RPC in the non-integrable system (Eq. (10) with Δ = 1 and J_2 = 0.5) after quantum quench from the Néel state. The black dots are the time average of $S_2(l,t)$ and the dotted line is the fitting of them by Eq. (5). The inset shows the dynamics of the 2REE at the center of the system.

File Name: Supplementary Movie 2

Description: **Dynamics of the second Rényi Page curve in an integrable system**. Dynamics of the 2RPC in the integrable system (Eq. (10) with Δ = 1 and J_2 = 0) after quantum quench from the Néel state. The black dots are the time average of $S_2(l,t)$ and the dotted line is the fitting of them by Eq. (5). The inset shows the dynamics of the 2REE at the center of the system.
