# Supplementary figures and images for: Universality in volume-law entanglement of scrambled pure quantum states
Source: Nat Commun. 2018 Apr 24;9:1635. doi: 10.1038/s41467-018-03883-9 (PMC5915398; doi:10.1038/s41467-018-03883-9)

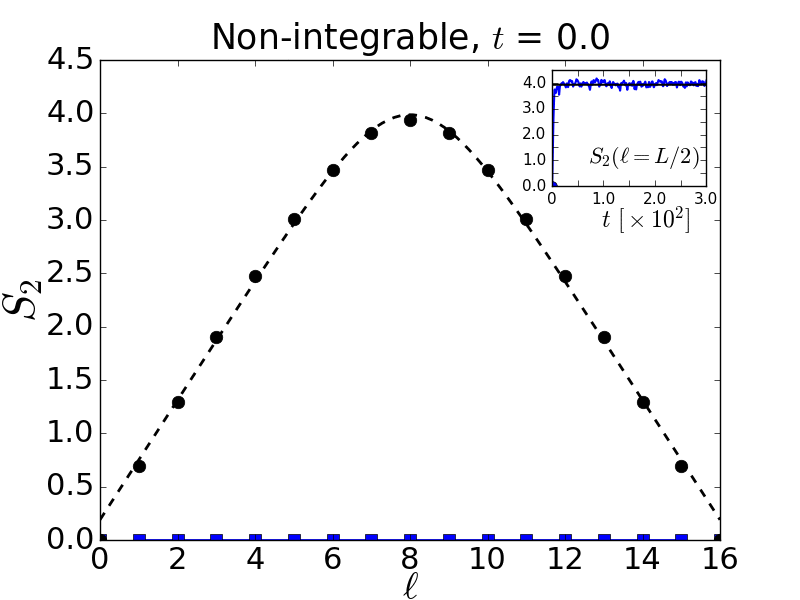

Supplement: Supplementary file 3 — Supplementary Movie 1 [file 41467_2018_3883_MOESM3_ESM.gif]

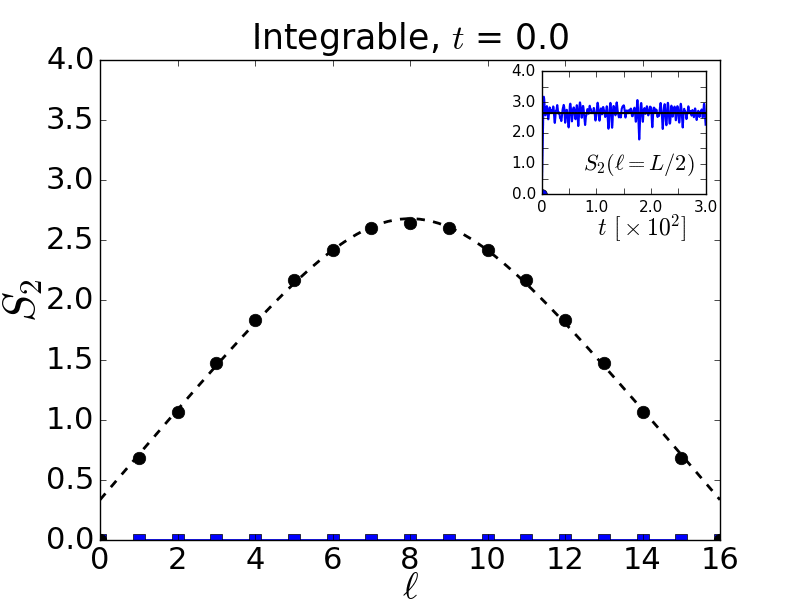

Supplement: Supplementary file 4 — Supplementary Movie 2 [file 41467_2018_3883_MOESM4_ESM.gif]
